# Premedication with reformulated simethicone and sodium bicarbonate improves mucosal visibility during upper gastrointestinal endoscopy: a double-blind, multicenter, randomized controlled trial

**DOI:** 10.1186/s12876-021-01623-w

**Published:** 2021-03-18

**Authors:** Xueqin Chen, Ning Dai, Yanyong Deng, Xin Sun, Mingqing Zhang, Jie Pan, Zhiming Huang, Guoliang Ye, Jianmin Si, Lan Wang, Shujie Chen

**Affiliations:** 1 Department of Gastroenterology, Sir Run Run Shaw Hospital Affiliated To Zhejiang University, 3 Qingchun Road East, Jianggan District, Hangzhou, Zhejiang China; 2 Department of Endoscopy Lab, Qingdao Municipal Hospital, 1 Jiaozhou Road, North District, Qingdao, Shangdong China; 3 Department of Gastroenterology, Affiliated Dongnan Hospital of Xiamen University, 269 Huazhong Road, Longwen District, Zhangzhou, Fujian China; 4 Department of Gastroenterology, Wenzhou City Second People′s Hospital, 32 Dajane Lane, Wenzhou, Zhejiang China; 5 Department of Gastroenterology, Wenzhou Medical University First Affiliated Hospital, Cai Village on the South White Elephant Street, Ouhai District, Wenzhou, Zhejiang China; 6 Ningbo City Third Hospital, 247 People′s Road, Jiangbei District, Ningbo, Zhejiang China

**Keywords:** Reformulated simethicone emulsion, Sodium bicarbonate, White flocculate precipitate, Esophagogastroduodenoscopy

## Abstract

**Background:**

The reformulated simethicone emulsion from Berlin Chemical AG might develop white flocculate precipitate covering the gastric mucosa when used before esophagogastroduodenoscopy (EGD). We aim to investigate whether combining the reformulated simethicone emulsion with 5% sodium bicarbonate solution could prevent the development of white precipitate and improve visibility during EGD.

**Methods:**

Our clinical study involved 523 patients. They were randomly assigned to two groups. In Group A, patients received a warm solution containing 30 ml 5% sodium bicarbonate solution and 15 ml reformulated simethicone emulsion. In Group B, patients received 45 ml 40 °C lukewarm water. Visibility scores were recorded and analyzed. Flushes, volume of flush water, overall time taken for EGD and complications during or after the procedure were also recorded.

**Results:**

We found that no white precipitate was observed during EGD in Group A. Moreover, visibility scores in Group A were significantly lower (*P* < 0.01). Patients in Group A had fewer flushes (*P* < 0.01) and smaller volume of flush water (*P* < 0.01). In addition, the overall time taken for the EGD procedure was significantly shorter in Group A (*P* < 0.01). The percentage of patients who had no adverse response was significantly higher in Group A than in Group B (*P* < 0.01).

**Conclusions:**

Premedication with a mixed solution of 15 ml reformulated simethicone emulsion and 30 ml 5% sodium bicarbonate solution can prevent the development of white precipitate, substantially enhancing mucosal visibility safely.

*Trial registration*: The registered name of the trial is “Efficacy of using premedication with reformulated simethicone emulsion during upper gastrointestinal endoscopy examination”. Its Current Controlled Trials number is ChiCTR1900021689. Its date of registration is 11 September 2019. Retrospectively registered, http://www.medresman.org.cn/uc/sindex.aspx.

**Supplementary Information:**

The online version contains supplementary material available at 10.1186/s12876-021-01623-w.

## Background

Esophagogastroduodenoscopy (EGD) is the most important method for diagnosing upper gastrointestinal disease [1]. High-quality EGD examination will significantly increase the diagnostic rate of upper gastrointestinal disease, especially early or subtle lesions.

The major technical obstacle lies in the presence of foam and mucus over the gastric mucosal surfaces, which often results in impaired visibility, prolonged examination time, aggravating discomfort for patients and even missed diagnoses of early or subtle lesions. Published data from institutions in the United Kingdom have shown that 10–14% of gastric cancer patients had undergone EGD in the preceding 3 years [2, 3]. Impaired visibility may be a leading factor for missed diagnoses on EGD [4]. Thus, the elimination of foams and mucus over gastric mucosa is essential before endoscopic examination. It is generally accepted that premedication with antifoam/mucus agents before EGD can improve operation visibility [5–7]. Premedication with pronase [7, 8], *N*-acetylcysteine [9] or simethicone [4] before EGD improves procedural visibility. In addition, combinations of different antifoam/mucus agents might be more effective than single agents [4]. Premedication before EGD with sedation has proven to be safe [7]. Nevertheless, the optimal quantity, density and time of premedication have not yet been established.

For this reason, in East Asian countries, especially Japan, premedication with an antifoam/mucus agent before EGD has become a standard procedure [6]. However, there is insufficient evidence that premedication with antifoam/mucus agents before EGD can improve the detection rate of early or subtle lesions. The safety of antifoam/mucus agents also remains to be clarified, which may be the main reason why they have yet to be widely utilized in the West. The ideal antifoam/mucus agent should have nontoxic side effects, strong tolerance and wide applicability to different patients [10].

Among the variety of antifoam/mucus agents on the market, simethicone is the most common option for endoscopy [7]. It is effective in eliminating foam and mucus by decreasing the surface tension of bubbles or foam [11]. Moreover, simethicone was proven effective in improving mucosal visibility when used as a premedication for EGD [4]. The latest Asian consensus on the standards of diagnostic upper endoscopy for neoplasia has strongly recommended premedication with simethicone before EGD, classifying it as a level A recommendation [12].

Berlin Chemical AG (Germany) is the largest producer of simethicone emulsion in the world. To improve the stability of the product, the company’s formula was optimized and upgraded in October 2016. The reformulated simethicone emulsion has had its raw materials upgraded to make the product more stable. Compared to the original product, the reformulated simethicone emulsion is better in the following three aspects: (1) the surface changes from emulsion into a milky liquid with low viscosity; (2) the shelf life is 6 months instead of 28 days; and (3) the emulsion can be kept at room temperature rather than 25 °C. Overall, the reformulated simethicone emulsion is more stable. However, it was found that the reformulated simethicone emulsion might develop a small amount of white flocculate precipitate covering the gastric mucosa when used before EGD, which will impair the observation on endoscopy. To eliminate any white precipitate, some endoscopists have adopted sodium bicarbonate mixed with the reformulated simethicone emulsion as premedication before EGD. In our laboratory studies, it was found that premedication with 5% sodium bicarbonate solution combined with the reformulated simethicone emulsion could eliminate white precipitate effectively under acidic conditions. However, there are no evidence-based data on the optimal usage method or dosage of sodium bicarbonate in patients. Therefore, we have endeavored to create a standard recommendation for the usage of sodium bicarbonate in combination with the reformulated simethicone emulsion prior to EGD.

The present study aims to determine whether premedication with the reformulated simethicone emulsion combined with sodium bicarbonate can prevent the development of white precipitate and substantially improve mucosal visualization in upper gastrointestinal endoscopy examination. We also examined its safety and its effectiveness in reducing the time taken for the procedure.

## Methods

### Vitro experiment

The experiment was based on the estimates that the average volume of fasting gastric acid is 50 ml and the theoretical maximum volume of gastric acid is 100 ml [13]. Hydrochloric acid solution with a pH of 1.04 made from concentrated hydrochloric acid was used to simulate gastric acid. We reduced the gastric acid volume, simethicone emulsion and sodium bicarbonate dosage to 1/5 in our in vitro model experiment. The following procedures were performed: different volumes of 5% sodium bicarbonate solution were added to 3 ml simethicone emulsion, and the mixtures were shaken well. Then the mixed solutions were added to centrifuge tubes containing 10 ml artificial gastric acid to observe the stability of the simethicone after 0 min, 15 min, 45 min, 60 min and 240 min. The pH value was measured at the reaction endpoint. The above procedure was also performed with 20 ml artificial gastric acid.

## Clinical research

### Patients

The clinical part of this study was conducted in six hospitals simultaneously. From March 2017 to March 2018, a total of 523 patients aged 18 to 75 years old were eligible for participation in the study. Exclusion criteria were a contraindication for EGD, upper gastrointestinal tract stricture and dysphagia, known allergy to the premedication, pregnancy or breastfeeding, a history of upper gastrointestinal surgery, participation in other clinical studies within the previous month, life-threatening gastrointestinal disease and refusal. Informed consent was obtained from all participants prior to the study.

### Premedication and endoscopic procedure

Patients were randomly assigned to two groups (A:B allocation ratio 2:1) by random computer-generated numbers before the endoscopy procedure. A total of 535 patients were enrolled in the study. Twelve patients were excluded for meeting the exclusion criteria: ten patients had a history of upper gastrointestinal surgery, and two patients did not meet the age criteria. The endoscopists and nursing staff were blinded to the medications administered before EGD. In Group A, patients received warm mixed solution containing 30 ml 5% sodium bicarbonate solution and 15 ml reformulated simethicone emulsion (Berlin-Chemie AG, Berlin, Germany) 30 min before EGD. In Group B, patients received 45 ml 40 °C lukewarm water 30 min before EGD. Patients in both groups were instructed to walk back and forth 3 times within the prescribed 10-m area. All patients were given the standard recommendation of at least 8 h of liquid and solid fasting before the procedure. They received 10 ml lidocaine hydrochloride mucilage 10 min before the endoscopy procedure. Nurses completed the whole premedication procedure in such a way that the patients were blinded to the premedication used.

Endoscopists who had 5 or more years of experience performed conventional EGD. The patients underwent the whole procedure without sedation or anesthesia. At the same time, a nurse was assigned to record the relevant data during and after EGD for subsequent analysis. The endoscopists and nurses who completed the operation were unaware of the group assignment.

**Fig. 1:**
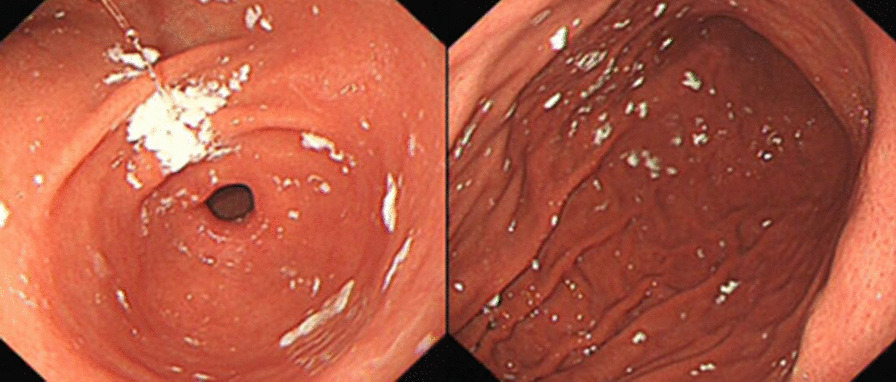
When using the reformulated simethicone emulsion before EGD, white flocculate precipitate developed and covered the gastric mucosa

**Fig. 2:**
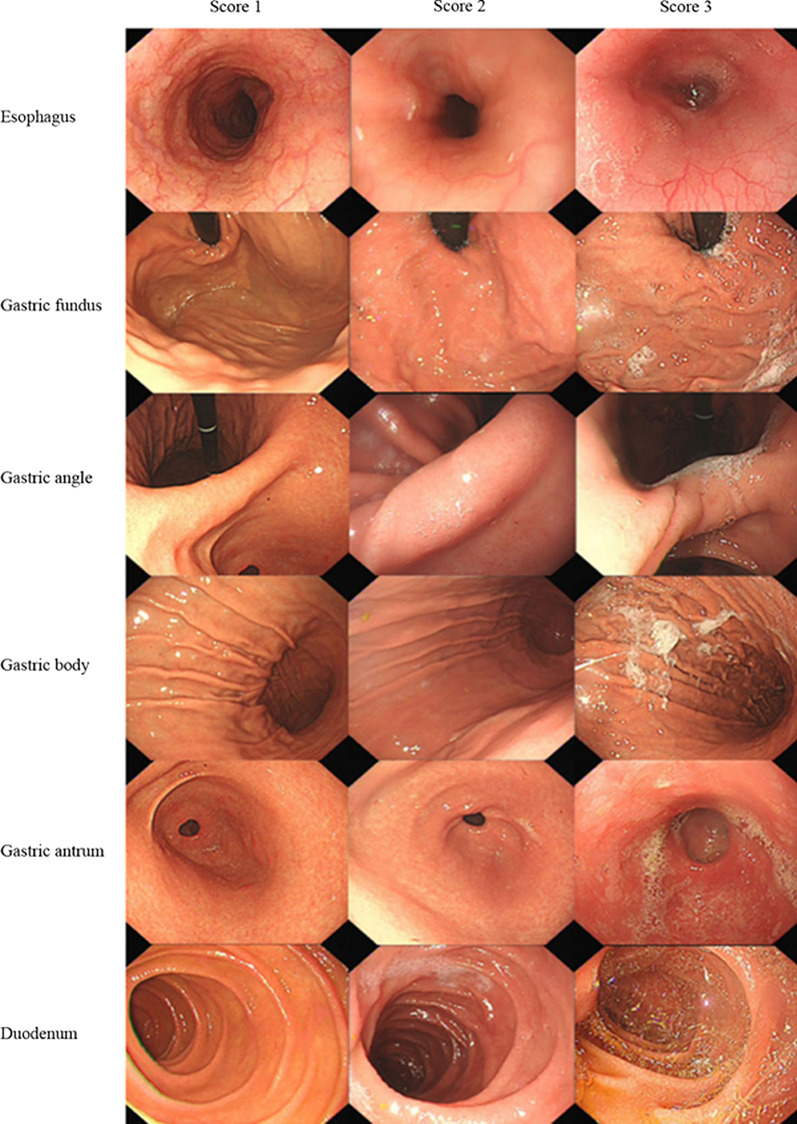
Criteria for the mucosal visibility score for each location: **a** Score 1, no adherent mucus and clear view of the mucosa; **b** Score 2, a thin coating of mucus without obscured vision; **c** Score 3, adherent mucus obscuring vision

**Fig. 3:**
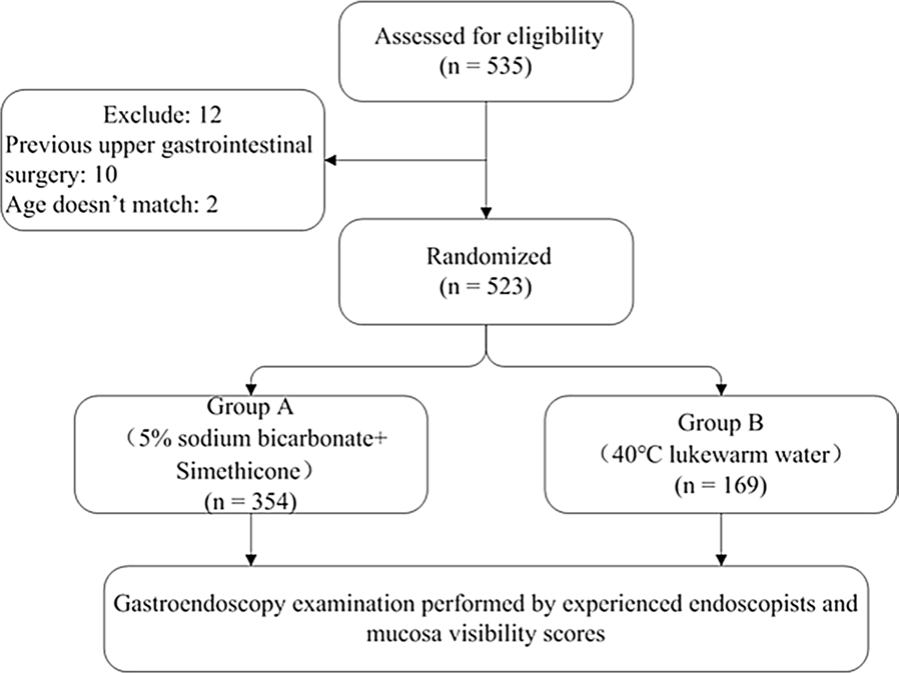
Flow chart of the present study

The whole operation was video-recorded. After the procedure, an experienced endoscopist in each hospital who had not participated in the examination reviewed the endoscopic videos and images. Both the endoscopists who performed the procedures and the EGD participants remained blinded to the premedication drugs used during the study. First, we evaluated whether white precipitate developed (Fig. 1). Subsequently, each patient was analyzed based on the following criteria. The primary criterion was mucosal visibility. Visibility scores of the esophagus, gastric fundus, gastric body, gastric antrum, gastric angle and duodenal mucosa were recorded. The scoring system is shown in Fig. 2: score 1, no adherent mucus and a clear view of the mucosa; score 2, a thin coating of mucus but no obscured vision; and score 3, adherent mucus obscuring vision [14]. The sum of the scores of all the sites made up the final score. The secondary criteria included the time taken for the whole procedure, the amount of saline water consumed for mucosal cleansing and adverse reactions during or after the procedure. We evaluated the occurrence of adverse reactions by asking whether the patient felt painful or not during the procedure and within an hour after the procedure. A flow chart of the present study is shown in Fig. 3.

### Statistical analysis

We adopted a study power of 90%, set the type I error as 0.05, and set the minimum expected difference in visibility scores between the two groups as 0.15, according to previous data [15], assuming a 15% missing rate. In the end, a total of 523 patients were included in the study.

Acquired data were interpreted as mean ± standard deviation (SD) or number (%). Continuous variables were assessed by Student’s t-test, while categorical variables were tested by the chi-squared test or Fisher’s exact test. *P* < 0.05 was considered statistically significant. Statistical analysis was carried out with SPSS 22.

## Results

### Investigation of the stability of the reformulated simethicone emulsion mixed with different doses of sodium bicarbonate solution under acidic conditions in vitro

When the reformulated simethicone emulsion was mixed with artificial gastric acid, white precipitate developed. When the pH of the reaction endpoint was higher than 5.5, there was no white precipitate in the in vitro experiment (Additional file 1: Appendix 1; Appendix 2). In 10 ml of artificial gastric acid, a 1-ml or 1.2-ml sodium bicarbonate solution mixed with 3 ml reformulated simethicone emulsion developed a large amount of precipitate. A 1.4-ml or 1.6-ml sodium bicarbonate solution mixed with 3 ml reformulated simethicone emulsion developed a small amount of precipitate. A 1.8-ml or 2-ml sodium bicarbonate solution mixed with 3 ml reformulated simethicone emulsion developed a very small amount of precipitate. At least 3 ml of 5% sodium bicarbonate solution mixed with 3 ml reformulated simethicone emulsion was needed to prevent the development of precipitate (Additional file 1: Appendix 1). In 20 ml of artificial gastric acid, 2 ml or 3 ml sodium bicarbonate solution mixed with 3 ml reformulated simethicone emulsion developed a large amount of precipitate. Sodium bicarbonate solution (3.5 ml or 4.0 ml) mixed with 3 ml reformulated simethicone emulsion developed a very small amount of precipitate. At least 5 ml of 5% sodium bicarbonate solution mixed with 3 ml reformulated simethicone emulsion was needed to prevent the development of precipitate (Additional file 1: Appendix 2). Based on the estimate that the average volume of fasting gastric acid is 50 ml and the theoretical maximum volume of gastric acid is 100 ml, a mixed solution containing 30 ml 5% sodium bicarbonate and 15 ml reformulated simethicone emulsion was chosen to conduct the clinical study.

### Demographic characteristics of patients

A total of 535 patients were identified and enrolled in the study, out of which twelve patients were excluded; therefore, 523 patients were included in the final analysis (Fig. 3). There was no significant difference between the demographic characteristics of the two investigated groups. There was also no difference in referral category or clinical indication between the two groups (Table 1).

**Table 1:** Demographic characteristics of enrolled patients

	Group A (n = 354)	Group B (n = 169)	*P *value
Age, mean ± SD (years)	48.5 ± 13.2	47.5 ± 12.7	0.487
Sex, n			1.000
Male	201	96	
Female	153	73	
Indication			0.338
No obvious symptom	68	37	
Screening	64	33	
* H. *plori infection	3	4	
Increased serum tumor marker	1	0	
Acid regurgitation	22	6	
Dyspepsia	182	79	
Abdominal pain or discomfort	62	40	
Vomit	1	0	
Constipation	0	1	
Dysphagia	1	0	
Gastrointestinal bleeding	10	4	
Distention	8	2	

### Premedication with a mixed solution of the reformulated simethicone emulsion and 5% sodium bicarbonate solution can enhance mucosal visibility

White precipitate was not observed during EGD in any patients in Group A. The visibility scores of Group A and Group B in the esophagus (1.12 ± 0.40 vs 1.53 ± 0.59, *P* < 0.01), gastric fundus (1.23 ± 0.48 vs 1.98 ± 0.73, *P* < 0.01), gastric angle (1.04 ± 0.22 vs 1.36 ± 0.56, *P* < 0.01), gastric body (1.39 ± 0.63 vs 2.05 ± 0.76, *P* < 0.01), gastric antrum (1.10 ± 0.34 vs 1.63 ± 0.70, *P* < 0.01), and duodenum (1.08 ± 0.30 vs 1.47 ± 0.58, *P* < 0.01) and the total visibility score (6.97 ± 1.39 vs 10.01 ± 2.47, *P* < 0.01) are shown in Table 2. Every score for Group A was significantly lower than that of Group B, indicating better mucosal visualization for Group A.

**Table 2:** Visibility scores

	Group A (n = 354)	Group B (n = 169)	*P *value
Esophagus	1.12 ± 0.40	1.53 ± 0.59	< 0.01
Gastric fundus	1.23 ± 0.48	1.98 ± 0.73	< 0.01
Gastric angle	1.04 ± 0.22	1.36 ± 0.56	< 0.01
Gastric body	1.39 ± 0.63	2.05 ± 0.76	< 0.01
Gastric antrum	1.10 ± 0.34	1.63 ± 0.70	< 0.01
Duodenum	1.08 ± 0.30	1.47 ± 0.58	< 0.01
Total score	6.97 ± 1.39	10.01 ± 2.47	< 0.01

### The influence of premedication with the reformulated simethicone emulsion and 5% sodium bicarbonate solution on the number of flushes and volume of flush water during EGD

There were significant differences in the number of flushes needed to clear stubborn mucus or bile between the two groups (Table 3). Significantly fewer flushes were required in Group A than in Group B (*P* < 0.01) in the gastric fundus, gastric angle, gastric body, gastric antrum, and duodenum, as well as the total number. The volumes of water needed to flush the esophagus, gastric fundus, gastric angle, gastric body, gastric antrum, duodenum and total esophagogastric tract were all significantly lower in Group A than in Group B (*P* < 0.01).

**Table 3:** Flushes and volume of flush water

	Group A(n = 354)	Group B(n = 169)	*P *value
Number of flushes			
Esophagus	0.05 ± 0.43	0.09 ± 0.33	0.271
Gastric fundus	0.16 ± 0.44	0.74 ± 0.93	< 0.01
Gastric angle	0.04 ± 0.24	0.17 ± 0.38	< 0.01
Gastric body	0.38 ± 0.77	1.12 ± 1.40	< 0.01
Gastric antrum	0.10 ± 0.37	0.60 ± 0.79	< 0.01
Duodenum	0.05 ± 0.22	0.28 ± 0.51	< 0.01
Total times	0.78 ± 1.36	3.01 ± 2.62	< 0.01
Volume of flush water(ml)			
Esophagus	0.79 ± 4.87	2.75 ± 10.07	< 0.01
Gastric fundus	5.11 ± 14.62	28.31 ± 39.15	< 0.01
Gastric angle	0.90 ± 7.04	3.96 ± 12.36	< 0.01
Gastric body	15.99 ± 35.33	42.91 ± 68.75	< 0.01
Gastric antrum	3.68 ± 15.11	19.76 ± 35.32	< 0.01
Duodenum	1.38 ± 6.35	5.80 ± 15.41	< 0.01
Total volume	27.86 ± 53.57	103.50 ± 128.03	< 0.01

### Time required for endoscopy examination

**Fig. 4:**
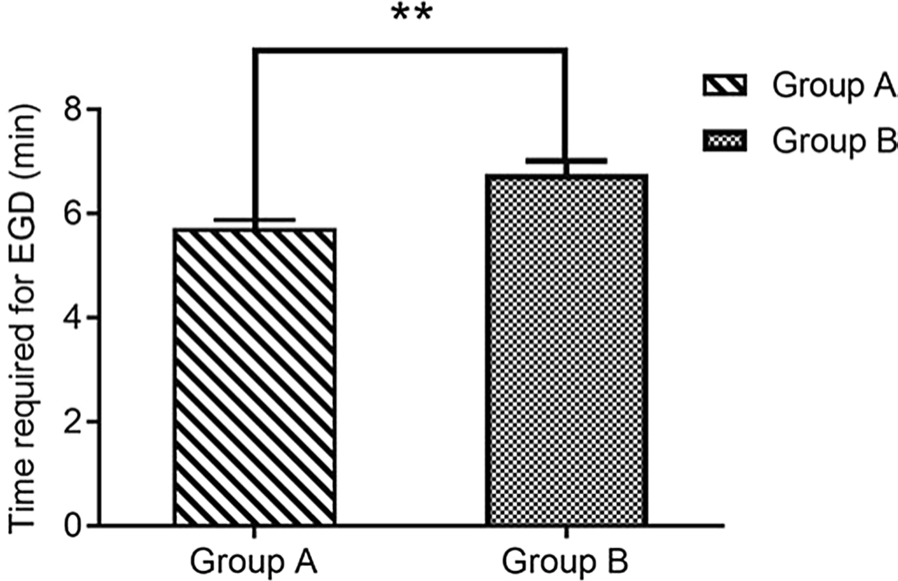
Time required for endoscopic examination (**P < 0.01). The mean overall time for gastroscopy in Group A was significantly shorter than that in Group B (*P* < 0.01)

Figure 4 shows that the mean overall time for gastroscopy in Group A (5.73 ± 2.65 min) was shorter than that in Group B (6.76 ± 3.13 min). The difference between the two groups (*P* < 0.01) was significant.

### The complications of premedication with a mixed solution of the reformulated simethicone emulsion and 5% sodium bicarbonate solution

**Fig. 5:**
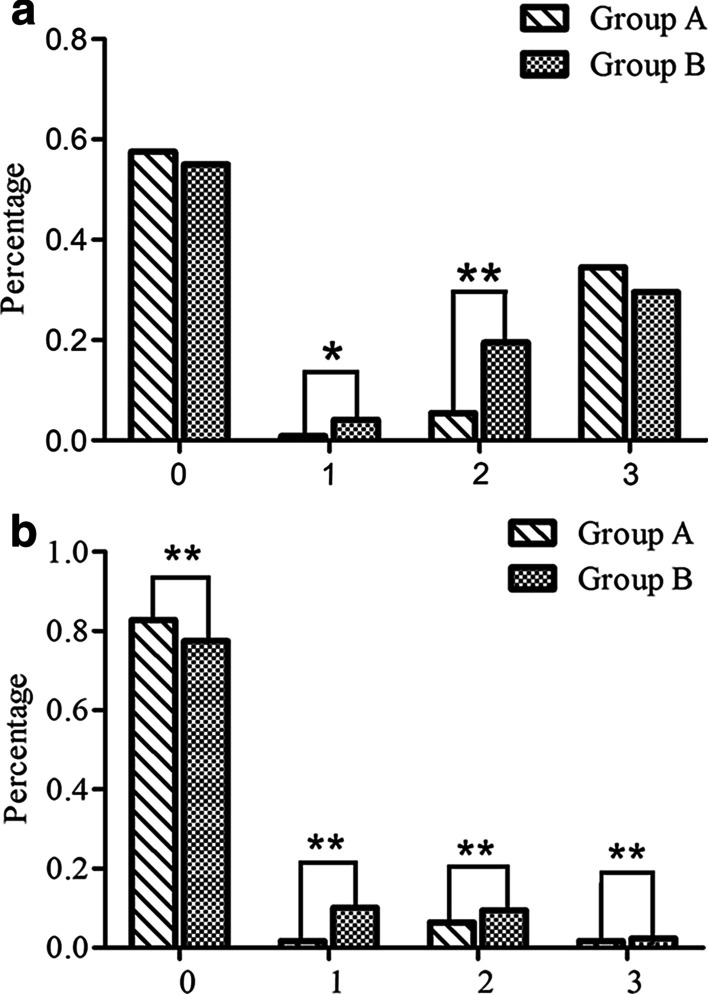
**a** Incidences of complications during EGD (0: No symptoms; 1: Abdominal pain; 2: Distension; 3: Nausea; *P < 0.05, **P < 0.01). The incidence rates for abdominal pain and distension during EGD were significantly higher in Group B than in Group A. However, there was no significant difference between Group A and Group B in the percentage of patients who had no adverse response or nausea during EGD. **b **Incidence of complications after EGD (0: No symptoms; 1: Abdominal pain; 2: Distension; 3: Nausea; **P < 0.01). Abdominal pain, distension, and nausea after EGD occurred significantly more often in Group B than in Group A (*P* < 0.01). In addition, the percentage of patients who had no adverse response after EGD was significantly higher in Group A (*P* < 0.01)

The incidence rates of abdominal pain (0.8% vs 4.1%, *P* < 0.05) and distension (5.4% vs 19.5%, *P* < 0.01) during EGD were significantly higher in Group B than in Group A (Fig. 5a). There was no significant difference between Group A and Group B in the percentage of patients who had no adverse response (57.6% vs 55.0%) or nausea (34.5% vs 29.6%) during EGD.

Abdominal pain (10.1% vs 1.7%), distension (9.5% vs 6.5%) and nausea (2.4% vs 1.7%) after EGD occurred significantly more often in Group B than in Group A (*P* < 0.01), as shown in Fig. 5b. In addition, the percentage of patients who had no adverse response after EGD (82.8% vs 77.5%) was significantly higher in Group A (*P* < 0.01).

## Discussion

Foams and bubbles can impair the detection of small and early lesions in EGD. Flushing during surgery may result in a longer procedure time and increase the risk of adverse reactions. Proper preparation can lower the need to flush the mucus during the procedure. It is widely accepted that premedication with an antifoam/mucus agent can improve endoscopic visibility during EGD [9]. Thus, the usage of antifoam/mucus agents before EGD may improve acceptance by patients. Simethicone has been used as an effective antifoam/mucus agent [15]. Recently, Berlin Chemical AG modified the formula of its simethicone emulsion. Clinical data have shown that a small amount of white flocculate precipitate still existed during EGD after premedication with the reformulated simethicone emulsion. In some hospitals, it was recommended that patients take sodium bicarbonate before the new simethicone emulsion. However, further evidence-based research is needed to determine the optimal method of administration and dosage of sodium bicarbonate with the reformulated simethicone emulsion. The present study is the first trial to investigate the effectiveness and safety of a mixed solution containing the reformulated simethicone emulsion and sodium bicarbonate prior to EGD.

In our laboratory experiment, it was observed that a sodium bicarbonate solution combined with the reformulated simethicone emulsion could prevent the development of white precipitate by neutralizing artificial gastric acid. The results showed that a mixed solution containing at least 5 ml 5% sodium bicarbonate solution and 3 ml reformulated simethicone emulsion was needed to maintain the stability of simethicone in 20 ml artificial gastric acid. Therefore, in clinical research, a mixed solution containing 30 ml 5% sodium bicarbonate solution and 15 ml reformulated simethicone emulsion was adopted as premedication before EGD according to the theoretical maximum volume of gastric acid (100 ml) [13].

Mucosal visibility is paramount in detecting subtle and early lesions [16]. This study showed that premedication with a mixed solution of the reformulated simethicone emulsion and 5% sodium bicarbonate solution could prevent the development of white precipitate during EGD, substantially enhancing mucosal visibility in Group A compared to the control group. Suvakovic et al*.*[3] analyzed 181 advanced gastric cancer patients and found that 11.2% had undergone gastroendoscopy that did not detect the cancer. It is likely that a number of things are to blame for the missed diagnoses by EGD, including patient preparation. To a large extent, the quality of preparation before EGD influences the quality of the operation. Proper preparation may help to decrease the missed diagnosis rate of EGD. Mucosal visibility, a core factor in evaluating the quality of EGD, is influential in the detection of lesions. Some previous studies showed that *N*-acetylcysteine or Pronase combined with simethicone could improve mucosal visibility [4, 9]. However, the increased cost is too significant to be ignored. Sodium bicarbonate solutions cost much less. Premedication with a simethicone emulsion and sodium bicarbonate solution only increases the cost slightly. The unit prices of simethicone emulsion and sodium bicarbonate solution (100 ml) are 48 RMB and 1.6 RMB, respectively, so compared with using simethicone alone, this combination only costs an extra 1.6 RMB. Hence, reformulated simethicone emulsion combined with sodium bicarbonate solution could be a highly efficient premedication for EGD. In addition, improved mucosal visibility may contribute to the early diagnosis of gastric diseases, including gastric neoplasms. Early gastric cancer patients have excellent future survival rates and quality of life. Sue Ling et al. reported that the 5-year survival rate for patients who were diagnosed with gastric cancer early reached 98% [17], whereas the prognosis after advanced-stage detection is dismal [18]. Moreover, the majority of early diagnoses are opportunistic [19]. Thus, high-quality EGD is vital for the diagnosis and prognosis of gastric cancer.

This study also found that the numbers of flushes and the volumes of flush water used in most areas decreased in patients who received premedication with the mixed solution. There was also good evidence of improved mucosal visibility in Group A. There was no significant difference between Group A and Group B in terms of the number of flushes needed for the esophagus. A possible explanation is that most mucus and foam in the esophagus can be eliminated through aspiration, but not flushing. This study also observed a significantly shorter operation time for EGD in Group A. Similarly, Lee et al. [14] found that it took significantly less time to complete EGD in patients receiving simethicone and Pronase before operation. However, the time required for the EGD procedure was significantly longer in the premedication group than in the control group in Zhang’s study [7]. They suggested raised suspicion of performers and requiring more time for the ingestion of retention liquid during EGD [7]. The better results in our present study may be due to the enhanced mucosal visibility and fewer flushes, which let us observe more suspicious areas more easily. Most endoscopic examinations are not done under sedation in China; hence, a shorter procedure time can reduce discomfort for patients, which will contribute to higher satisfaction with EGD and avoid delayed diagnoses of gastric cancer [9].

EGD is an invasive operation. Many patients refuse it for fear of discomfort. In addition, some rare complications may lead to the need for surgery. In Japan and other East Asian countries, premedication before EGD is routine. Despite this, there is little evidence of the safety of using premedication before EGD in Western countries [16]. Our study investigated the safety of a premedication that included the reformulated simethicone emulsion combined with sodium bicarbonate solution. The results showed that the percentage of patients who had no adverse response was significantly higher in patients who had received the premedication before EGD. The incidence of abdominal pain and distension was significantly lower in Group A both during and after EGD. The percentage of patients who had nausea after EGD was also lower in Group A. These findings indicate that premedication with a mixed solution of the reformulated simethicone emulsion and sodium bicarbonate solution could reduce complications. Simethicone is commonly used for gas and distension [20]. It contributes to the relief of symptoms caused by the injection of gas during EGD. In addition, fewer flushes and smaller volumes of flush water result in less discomfort, which may reduce complications during and after gastroscopy operations.

There are some limitations to this study. We cannot cite published data showing that the reformulated simethicone actually causes worse visualization when used as a premedication before EGD. This study is a multicenter effort. In total, 34 endoscopists from six different hospitals were involved in this research. They performed gastroendoscopies in both groups at their respective centers, but we did not analyze how the outcomes varied by endoscopist. Additionally, we did not present any pathological information. The ability to detect diminutive lesions or early lesions is vital for the diagnosis of gastric cancer. However, it will take a long time to follow up a large enough number of patients to acquire conclusive results. In-depth investigation will be done in the future.

Our research is the first multicenter, prospective, double-blind, randomized controlled study to show the effectiveness and safety of premedication with a mixed solution of the reformulated simethicone emulsion and 5% sodium bicarbonate solution before EGD. The results showed that reformulated simethicone emulsion combined with 5% sodium bicarbonate solution can prevent the development of white precipitate and markedly enhance mucosal visibility. In addition, it has been proven to be safe and can reduce complications. As there is no standard recommendation or guideline for premedication with the reformulated simethicone emulsion and sodium bicarbonate solution, we recommend the routine use of premedication with a mixed solution of 15 ml reformulated simethicone emulsion and 30 ml 5% sodium bicarbonate solution 30 min before EGD.

## Conclusions

The reformulated simethicone emulsion might develop white flocculate precipitate covering the gastric mucosa when used before EGD. Premedication with a mixed solution of 15 ml reformulated simethicone emulsion and 30 ml 5% sodium bicarbonate solution can prevent the development of white precipitate, substantially enhancing mucosal visibility safely.

## Supplementary information

**Additional file 1:** **Appendix 1**. Stability of mixed solution contained simethicone and different doses of 5% sodium bicarbonate solution when added to artificial gastric acid (10mL). **Appendix 2**. Stability of mixed solution contained simethicone and different doses of 5% sodium bicarbonate solution when added to artificial gastric acid (20mL).

## Data Availability

The datasets used and/or analyzed during the current study are available from the corresponding author on reasonable request.

## Abbreviations

EGD: Esophagogastroduodenoscopy
SD: Standard deviation

## Statement

The current study adheres to CONSORT guidelines.
