# Supplementary material for: Premedication with reformulated simethicone and sodium bicarbonate improves mucosal visibility during upper gastrointestinal endoscopy: a double-blind, multicenter, randomized controlled trial
Source: BMC Gastroenterol. 2021 Mar 18;21:124. doi: 10.1186/s12876-021-01623-w (PMC7977252; doi:10.1186/s12876-021-01623-w)
Supplement: Supplementary file 1 — Additional file 1: Appendix 1. Stability of mixed solution contained simethicone and different doses of 5% sodium bicarbonate solution when added to artificial gastric acid (10mL). Appendix 2. Stability of mixed solution contained simethicone and different doses of 5% sodium bicarbonate solution when added to artificial gastric acid (20mL). [file 12876_2021_1623_MOESM1_ESM.doc]

**Appendix 1** Stability of mixed solution contained simethicone and different doses of 5% sodium bicarbonate solution when added to artificial gastric acid (10mL)

| Group | Sodium bicarbonate  （mL） | Artificial gastric acid（mL） | Simethicone（mL） | pH of Reaction Endpoint | Status of simethicone | | | |
| --- | --- | --- | --- | --- | --- | --- | --- | --- |
|  |  |  |  |  | 0min | 30min | 60min | 240min |
| 1 | 1 | 10 | 3 | <2.0 | ↓↓↓ | ↓↓↓ | ↓↓↓ | ↓↓↓ |
| 2 | 1.2 | 10 | 3 | 2 | ↓↓↓ | ↓↓↓ | ↓↓↓ | ↓↓↓ |
| 3 | 1.4 | 10 | 3 | 3.5 | ↓ | ↓ | ↓ | ↓ |
| 4 | 1.6 | 10 | 3 | 4.5-5.0 | ↓ | ↓ | ↓ | ↓ |
| 5 | 1.8 | 10 | 3 | 5.0-5.5 | ↓- | ↓- | ↓- | ↓- |
| 6 | 2 | 10 | 3 | 5.0-5.5 | ↓- | ↓- | ↓- | ↓- |
| 7 | 3 | 10 | 3 | 5.5-6.0 | -- | -- | -- | -- |
| 8 | 6 | 10 | 3 | 6 | -- | -- | -- | -- |

↓↓↓: a large amount of precipitation, ↓↓: some precipitation, ↓: a small amount of precipitation, ↓-: very small amount of precipitation --: no precipitation

**Appendix 2** Stability of mixed solution contained simethicone and different doses of 5% sodium bicarbonate solution when added to artificial gastric acid (20mL)

| Group | Sodium bicarbonate  （mL） | Artificial gastric acid（mL） | Simethicone（mL） | pH of Reaction Endpoint | Status of simethicone | | | |
| --- | --- | --- | --- | --- | --- | --- | --- | --- |
|  |  |  |  |  | 0min | 30min | 60min | 240min |
| 1 | 2 | 20 | 3 | <2.0 | ↓↓↓ | ↓↓↓ | ↓↓↓ | ↓↓↓ |
| 2 | 3 | 20 | 3 | 3.5 | ↓↓↓ | ↓↓↓ | ↓↓↓ | ↓↓↓ |
| 3 | 3.5 | 20 | 3 | 5.0-5.5 | ↓- | ↓- | ↓- | ↓- |
| 4 | 4 | 20 | 3 | 5.5 | ↓- | ↓- | ↓- | ↓- |
| 5 | 5 | 20 | 3 | 6 | -- | -- | -- | -- |

↓↓↓: a large amount of precipitation, ↓↓: some precipitation, ↓: a small amount of precipitation, ↓-: very small amount of precipitation --: no precipitation
